# The Role of Intraoperative Flow Cytometry in Surgical Oncology: A Systematic Review

**DOI:** 10.3390/cancers17243898

**Published:** 2025-12-05

**Authors:** Eleni Romeo, Georgios S. Markopoulos, George Vartholomatos, Spyridon Voulgaris, George A. Alexiou

**Affiliations:** 1 Department of Neurosurgery, University Hospital of Ioannina, School of Medicine, S. Niarhou Avenue, 45500 Ioannina, Greece; e.romeo@uoi.gr (E.R.); svoulg@otenet.gr (S.V.); 2 Neurosurgical Institute, University of Ioannina, 45500 Ioannina, Greece; 3 Haematology Laboratory, Unit of Molecular Biology and Translational Flow Cytometry, 45110 Ioannina, Greece; geomarkop@gmail.com (G.S.M.); gvarthol@gmail.com (G.V.); 4 Laboratory of Physiology, Faculty of Medicine, University of Ioannina, 45500 Ioannina, Greece

**Keywords:** intraoperative, flow cytometry, glioma, meningioma, margins, head and neck tumors, breast cancer, colorectal cancer

## Abstract

**Purpose:** The aim of this review is to evaluate the role of intraoperative flow cytometry (IFC) in tumor surgery. **Methods:** The Medline, Scopus, and Cochrane databases were searched up to 21 June 2025 to identify all available studies that met the inclusion criteria for final evaluation. To assess the risk of bias and applicability concerns, the Quality Assessment of Diagnostic Accuracy Studies 2 (QUADAS-2) tool was used. **Results:** A total of 22 studies involving 1511 patients with various tumor types were analyzed to assess the utility of IFC in the rapid diagnosis of tumors. The studies investigated IFC’s role in tumor grading, margin delineation, prognostic evaluation, and in differentiating neoplastic from benign lesions, as well as normal from cancerous tissues. In brain tumors, particularly gliomas and meningiomas, IFC demonstrated high diagnostic performance, with reported sensitivities ranging from 61% to 100% and specificities from 66% to 100%. Studies on non-brain tumors also showed high accuracy in distinguishing neoplastic from normal tissues, with sensitivities and specificities exceeding 85% in most cases. The most promising results were observed in brain tumor surgery, although its application in other tumor types continues to expand. **Conclusions:** IFC appears to be a valuable intraoperative tool in surgical oncology, providing rapid results within minutes and assisting in surgical and therapeutic decision-making. Nonetheless, studies remain limited, and further research is needed, particularly for non-brain tumors, to establish standardized cut-off values and enhance diagnostic reliability.

## Simple Summary

This review examines the role of intraoperative flow cytometry (iFC) as a rapid tool for cancer diagnosis and for guiding surgeons’ decisions in the operating room. By evaluating 22 studies involving more than 1500 patients, we found that this method can reliably distinguish cancerous from non-cancerous tissue, determine tumor grade, and help define tumor margins. Its best performance has been reported in brain tumor surgery, where accuracy is particularly high, but research in other cancers such as breast, head and neck, and colorectal tumors is steadily increasing. Although results are promising, more studies are needed to standardize the technique and confirm its usefulness across different tumor types.

## 1. Introduction

Flow cytometry (FC) is a well-established technology used in the diagnosis of hematological malignancies. It is a laser-based, gold-standard method for determining the cell cycle fraction. FC enables rapid, multi-parametric analysis of cell populations and is employed in the diagnosis of various pathologies, including malignancies, infections, immune system disorders, and others. Cells or particles analyzed using flow cytometry are assessed based on visible light scatter and one or more fluorescence parameters. Samples are prepared for fluorescence analysis by transfecting cells to express fluorescent proteins (e.g., GFP), staining with fluorescent dyes (such as Propidium Iodide for DNA), or using fluorescently labeled antibodies (e.g., CD3-FTIC) [1]. FC offers high reproducibility in cell analysis through specialized data analysis software. Expert interpretation is used to guide therapy and to monitor disease progression and follow-up in various conditions [2].

Cancer is a leading cause of morbidity and mortality globally, accounting for 19.8% of all deaths in the United States in 2023 [3]. Early diagnosis and treatment of cancer can provide patients with the opportunity for better overall survival and improved outcomes. In surgical oncology, the use of techniques that can distinguish malignant from normal tissue plays a critical role. These approaches facilitate real-time diagnosis, enable high-precision tumor resection, and contribute substantially to achieving optimal surgical results. Over the past decade, a novel intraoperative method, intraoperative flow cytometry (IFC), has been developed as a useful tool for cancer diagnosis and treatment, particularly in central nervous system (CNS) tumors [4,5]. Beyond CNS tumors, this technique has been further developed and is now applied more broadly across a wide range of tumors, including those of the head and neck, breast, gynecological, gastrointestinal, primary and secondary liver, pancreatic, bladder, and skin cancers [6,7,8,9,10,11,12,13,14,15].

IFC offers several advantages as a diagnostic tool in oncology, providing rapid, quantitative, and multiparametric cell analysis using minimal tissue [2]. It delivers objective data on cell cycle distribution, DNA ploidy, and antigen expression with high sensitivity and reproducibility, making it suitable for time-sensitive intraoperative applications [16]. IFC is used in surgical oncology to analyze cells labeled with specific antibodies and cluster of differentiation (CD) markers, as well as to assess DNA ploidy and cell cycle phases (G0/G1, S, and G2/M) [16]. The G0/G1 phase represents the initial stage of the cell cycle, where cells either prepare for DNA replication or enter a quiescent state (G0). Cells in the S phase synthesize DNA, while those in the G2/M phase divide to produce two daughter cells. By staining fresh tissue samples with dyes such as propidium iodide, IFC quantifies the proportion of cells in each phase. To enhance intraoperative tumor characterization, indices such as the malignancy index (MI) and tumor index (TI) are used. The TI, calculated from the combined percentages of cells in the S and G2/M phases, reflects tumor proliferative activity [6], whereas the MI (or proliferation index, PI) represents the ratio of proliferating and/or aneuploid cells to the total cell population [5,17,18]. Additionally, IFC can determine the DNA index (DI), which compares DNA content between normal diploid cells and tumor cells; a DI of 1.0 indicates a normal diploid population [2].

Today, the IFC has been increasingly investigated in brain surgery and other tumor types as a potentially valuable tool for rapid intraoperative diagnosis and guidance during tumor resection. The primary aim of this systematic review is to evaluate the diagnostic accuracy, clinical utility, and intraoperative feasibility of IFC across different oncologic surgeries. The secondary objectives are to assess the role of IFC in tumor grading, margin delineation, and prognostic evaluation, and to identify methodological limitations and knowledge gaps in the existing literature that may guide future efforts toward standardization and broader clinical implementation of this technique. Importantly, no prior systematic review has comprehensively examined IFC across the full spectrum of oncologic surgeries or compared its diagnostic performance among different types of cancer. The present review is the first to synthesize all available clinical evidence on IFC applications in surgical oncology, providing an integrated overview of its diagnostic accuracy, clinical feasibility, and potential role in guiding intraoperative decision-making.

## 2. Material and Methods

The present systematic review was conducted using Preferred Reporting Items for Systematic Reviews and Meta-analyses (PRISMA) 2020 guidelines [19]. This systematic review was not prospectively registered in PROSPERO or any other international registry. The decision not to register was due to the fact that the review process was initiated and completed before submission, and retrospective registration is not permitted by PROSPERO. Nevertheless, the review was conducted in accordance with the PRISMA 2020 guidelines, ensuring transparency, methodological rigor, and reproducibility throughout all stages of literature search, selection, and synthesis [19].

### 2.1. Search Strategy

We conducted a systematic search to retrieve relevant studies evaluating the effectiveness of IFC in the intraoperative characterization of tumor types and margins in surgical oncology. The Medline, Cochrane, and Scopus databases were searched using the following query: [(intraoperative flow cytometry) AND ((brain tumors) OR (intracranial tumors) OR (meningiomas) OR (gliomas) OR (central nervous system lymphomas) OR (brain metastasis) OR (glioblastomas) OR (cancer) OR (tumor) OR (tumor margins) OR (breast cancer) OR (pancreatic cancer) OR (kidney cancer) OR (cancer detection))]. The last search in all databases was performed on 1 June 2025.

### 2.2. Inclusion and Exclusion Criteria

Studies were included if they met the following criteria: (1) evaluated the use of IFC in patients undergoing tumor surgery; (2) utilized IFC for tumor type differentiation, malignancy grading, and/or delineation of tumor margins to enhance intraoperative guidance and resection, and reported clinical outcomes or diagnostic accuracy parameters; (3) included human subjects; and (4) were original research articles conducted in any global setting (randomized controlled trials, cohort studies, case–control studies, or letters to the editor presenting new case series or data). Due to the limited number of available clinical studies on IFC, especially in non-brain tumors, broad inclusion criteria were applied to capture all relevant evidence. Letters to the editor presenting original data were included when they provided unique patient information or methodological details not available elsewhere. This approach aimed to ensure a comprehensive overview of current clinical applications while maintaining transparency regarding data sources.

Exclusion criteria comprised studies in which flow cytometry was not performed intraoperatively, as well as gray literature, conference abstracts, review articles, study protocols, book chapters, and non-English publications, to ensure methodological consistency and reproducibility.

### 2.3. Study Selection

Two independent reviewers (ER, GM) screened titles and abstracts for eligibility. Full-text articles were then assessed for inclusion. Disagreements were resolved by consensus or consultation with a third reviewer (GA).

### 2.4. Data Extraction

Data from eligible studies were collected in a standardized table pre-approved by all authors prior to extraction. The table was independently completed by two authors (ER and GA) to ensure consistency and minimize bias. Extracted data included study characteristics (DOI, first author, year of publication), study design (prospective or retrospective), IFC parameters assessed, tumor types, patient age range, total number of patients, diagnostic accuracy measures (sensitivity, specificity), and outcomes.

### 2.5. Risk of Bias and Applicability Assessment

The methodological quality and risk of bias of included diagnostic accuracy studies were assessed independently by two authors using the Quality Assessment of Diagnostic Accuracy Studies 2 (QUADAS-2) tool. Any disagreements were resolved through discussion or consultation with a third reviewer [20,21].

### 2.6. Data Synthesis

A qualitative synthesis of the findings was performed due to heterogeneity in study designs, tumor types, and outcome measures. Data evaluated included study population characteristics (gender, age, sample size), year of study, IFC parameters analyzed, tumor types assessed, study outcomes, performance metrics such as accuracy, specificity, sensitivity, AUC, and cut-off values if available.

No formal heterogeneity analysis (such as meta-regression or subgroup testing) was performed because of the qualitative nature of this review and the variability among included studies. Instead, observed differences in findings were summarized descriptively. Studies were organized into two main groups—brain tumor and non-brain tumor applications—and their diagnostic or prognostic outcomes were compared narratively. Additionally, a summary table was prepared to present all available cut-off values and thresholds reported across studies to illustrate the range and consistency of findings among tumor types.

## 3. Results

### 3.1. Search Findings

After the search, a total of 317 studies were identified through the MEDLINE/PubMed, Cochrane, and SCOPUS databases, with 242, 24, and 51 records, respectively. Following the removal of 36 duplicates, 281 studies remained and were screened by title and abstract, resulting in the exclusion of 87 studies. The full texts of the remaining 196 studies were retrieved and assessed for eligibility. A total of 124 studies were excluded because they did not use intraoperative flow cytometry. Twenty-one studies were excluded due to lack of accuracy, as they were case reports, letters to the editor without new data, or small case series. One study was excluded because it was a study protocol, and five studies were excluded because they reviewed articles. Finally, twenty-four studies were excluded for other reasons: three did not include human subjects, one did not exclusively use IFC, one had overlapping study populations with another study, one did not include tissue samples, one did not perform real-time IFC, thirteen were book chapters, and four did not use IFC rapid protocol.

**Figure 1:**
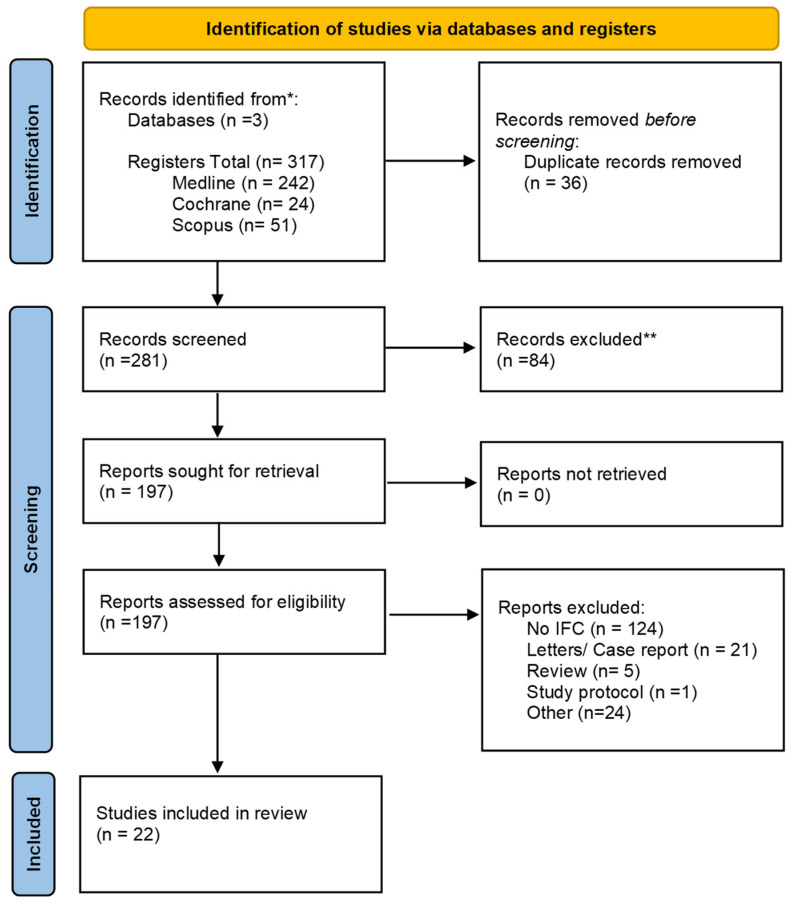
Flow diagram depicting systematic search using PRISMA guidelines for a systematic review. Source: Page MJ et al. BMJ 2021;372: n71. doi: 10.1136/bmj.n71 [19]. * Consider, if feasible to do so, reporting the number of records identified from each database or register searched (rather than the total number across all databases/registers). ** If automation tools were used, indicate how many records were excluded by a human and how many were excluded by automation tools.

Thus, 22 studies [4,5,6,8,9,10,11,12,14,15,18,22,23,24,25,26,27,28,29,30,31,32] were included in our review, encompassing 1511 patients who underwent tumor surgery with intraoperative IFC guidance. The search results and study-selection process are depicted in a PRISMA 2020 flow diagram (Figure 1), which outlines searches conducted across databases, registers, and other sources [19] (Table S1).

### 3.2. Risk of Bias and Applicability Assessment

A total of 22 studies were assessed for the presence of risk of bias in four domains using QUADAS-2. In the domain of patient selection, 11 studies [6,8,11,14,15,22,23,24,25,26,27] had low risk of bias, 7 studies [4,5,9,10,18,28,29] had high risk, and 4 studies [12,30,31,32] had unclear risk. Regarding the risk of bias in the index test, 3 studies [22,28,29] had high risk, 17 studies [4,5,8,9,10,11,14,15,18,23,24,25,26,27,30,31] had low risk, and 3 studies [12,32] had unclear risk. In the reference standard domain, only 2 studies [28,29] had high risk, 19 studies [4,5,6,8,9,10,12,14,15,18,22,23,24,25,26,27,30,31,32] had low risk, and 1 study had [11] unclear risk. Additionally, in the flow and timing domain, 17 studies [4,5,6,8,9,10,14,15,18,22,24,25,26,27,30,31,32] were assessed to have low risk, 1 study [29] had high risk, and 4 studies [11,12,23,28] had unclear risk.

Using the QUADAS-2 tool, concerns regarding applicability were evaluated across three domains. In the index test domain, 14 studies [6,8,11,14,15,22,23,24,25,26,27,30,31,32] had low risk, 7 studies [4,5,9,10,18,28,29] had high risk, and 1 study [12] had unclear risk. In the index test domain, low concerns of applicability were found in 18 studies [4,5,8,9,10,11,14,15,18,23,24,25,26,27,30,31,32], high risk in 3 studies [22,28,29,31], and unclear risk in 1 study [12]. In 2 studies [29,30], there were high concerns of applicability in the reference standard domain, while in 20 studies [4,5,6,8,9,10,12,14,15,18,22,23,24,25,26,27,31,32], there were low concerns in this domain, and 1 study [11] was unclear.

**Figure 2:**
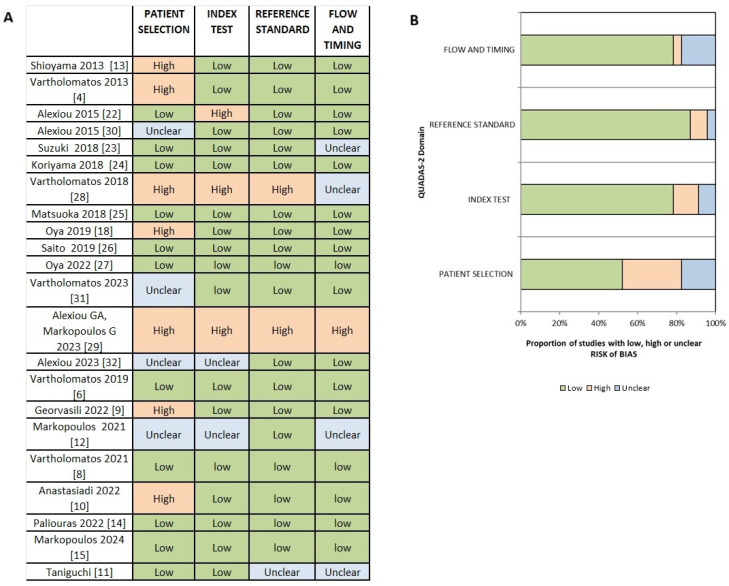
Summary of the risk of bias assessment for each study, presented as (**A**) a table and (**B**) a graph. Source: [4,6,8,9,10,11,12,13,14,15,18,22,23,24,25,26,27,28,29,30,31,32].

**Figure 3:**
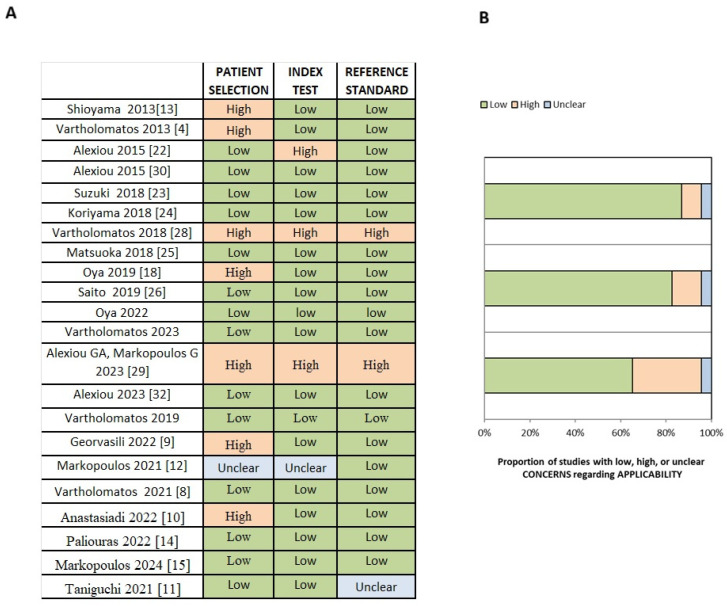
Summary of the applicability concerns for each study, presented as (**A**) a table and (**B**) a graph. Source: [4,6,8,9,10,11,12,13,14,15,18,22,23,24,25,26,27,28,29,30,31,32].

The results from the assessment of risk of bias and concerns of applicability are summarized in Figure 2 and Figure 3.

### 3.3. Characteristics of the Included Studies

Among the 22 studies that had been included in the systematic review study, 10 were prospective [5,6,8,10,14,15,18,25,27,30], 8 were retrospective [9,11,22,23,24,26,31,32], and in 4 studies [4,12,28,29], the type of study design was not available. A total of 1511 patients were included in this review, based on 22 studies published between 2013 and 2024, with sample sizes ranging from 5 to 250 patients per study. The patient populations included in the studies encompassed all age groups, with a mean age of 55.64 years. Of the 22 studies, 6 did not report the average age of participants [4,12,14,22,28,29], and 1 study provided only the age range (20–78 years) [31]. One study focused exclusively on the pediatric population, reporting a mean age of 5.1 years and an age range of 3 months to 14 years [24]. Additionally, one study included both pediatric (patients under 18 years of age) and adult populations [6,29].

Most studies focused on primary brain tumors (*n* = 14), followed by gastrointestinal and liver cancers (*n* = 3). The remaining studies investigated gynecological, breast, head and neck, bladder, and non-melanoma skin cancers. The applications of IFC across these studies included the following: prediction of tumor grade [4,5,22,25,30,31,32], assessment of tumor margins [5,8,9,10,15,30,31], differentiation between normal and neoplastic tissue [5,9,10,14,22,30], distinction between benign and neoplastic lesions [6], evaluation of prognosis and potential for tumor recurrence [4,18,23,27,28], and classification of specific tumor subtypes [11,12,15,22,24,28,29].

In most of the studies, the protocol was based on cell cycle fractions, including G0/G1, S phase, and G2/M phase, along with the calculation of the malignancy index (MI) or proliferation index (PI), which represents the number of aneuploid cells with an abnormal number of chromosomes relative to the total cell count, and the tumor index (TI), which combines the S phase and mitosis phase fractions [4,5,6,8,9,10,11,12,14,15,18,22,24,25,26,27,30,31,32]. In two studies, the protocol involved intraoperative immunophenotypic analysis to characterize specific cluster of differentiation (CD) markers in lymphoproliferative lesions [28,29]. Three studies focused on identifying the presence of aneuploidy [13,23,31], and two studies employed the DNA index (DI) [11,12]. The characteristics of the included studies are summarized in Table 1.

### 3.4. Qualitative Synthesis

The included studies collectively demonstrated that IFC offers a reliable and rapid method for analyzing tumor biology in real time, with strong applicability in both intracranial and other types of tumors. The outcomes and results from the studies included in this systematic review are summarized and analyzed in the following tables, organized according to tumor grading (Table 2), margin assessment (Table 3), prognostic evaluation (Table 4), and cut-off values (Table 5).

#### 3.4.1. Intracranial Tumors

Most of the studies in this review focused on primary brain tumors and mainly gliomas and meningiomas. The key IFC parameters that had been used included MI or PI, TI, DNA ploidy, and cell cycle fractions.

*Tumor grade*

For distinguishing the degree of malignancy or determining the presence of normal versus cancerous tissue, the sensitivity of IFC ranged from 61.4% to 94.1%, and specificity ranged from 66.7% to 100% [4,5,22,25,30,31,32]. Cell cycle fraction analysis was the most commonly used parameter for tumor grading, with various cut-off values reported.

In the comparison between low-grade gliomas (LGGs) and high-grade gliomas (HGGs), Vartholomatos et al. (2013) reported a G0/G1 phase cut-off of 78% and an S-phase cut-off of 4.3%, achieving sensitivities of 82.2% and 94.1%, respectively, and a specificity of 100% [4]. Similarly, Alexiou et al. (2015) found that HGGs had a lower G0/G1 phase fraction, with 75% being the optimal cut-off, resulting in 92.3% sensitivity and 91.7% specificity [30]. They also reported that in HGG, the G2/M phase exceeded 6% and the S-phase exceeded 9.7%, with sensitivities of 84.6% and 92.3% and specificities of 91.7% and 66.7%, respectively. These results indicate that cell-cycle parameters, particularly reductions in the G0/G1 fraction and increases in the S- and G2/M phases, correlate strongly with tumor grade and proliferative potential.

A 2023 study by Alexiou et al. further confirmed that higher-grade meningiomas exhibited lower G0/G1 fractions and higher S-phase and mitotic fraction [32]. In the pediatric population, one study reported a G0/G1 cut-off value of 89% and a mitotic fraction of ≤2%, both achieving 100% sensitivity and specificity in order to distinguish neoplastic lesions. A 2.2% cut-off for the S-phase was also proposed, with 98.4% sensitivity and 100% specificity. Additionally, a G0/G1 threshold of 81% was suggested in order to differentiate between low- and high-grade tumors, with 80% sensitivity and 88.9% specificity [22].

The MI and TI were also used for tumor grading, although they generally showed lower sensitivity and specificity compared to cell cycle fractions [5,25]. Shioyama et al. noted that LGGs exhibited lower MI values than HGGs [5]. In order to distinguish between Grade I and Grade II/III meningiomas, Matsuoka et al. reported an MI cut-off of 8%, with 64.7% sensitivity and 85.0% specificity [25]. Alexiou et al. used a TI cut-off of 11.4%, achieving 90.2% sensitivity and 72.2% specificity [32]. Finally, Vartholomatos et al. (2023) proposed a TI cut-off value of 17% for differentiating HGG from LGG and noted that all HGGs in their study were aneuploid, based on DNA index analysis [31].

Overall, these studies demonstrate that cell cycle fractions provide the highest diagnostic accuracy for tumor grading, while MI and TI offer complementary but less precise indicators of malignancy.

*Tumor margins*

One of the most clinically significant applications of IFC is its ability to delineate tumor margins in real time because it can be really helpful in achieving gross total resection. Several studies investigated the utility of various IFC parameters (MI, TI, DNA index, and cell cycle fraction), for identifying tumor infiltration at the margins [5,30,31].

In glioma patients, Shioyama et al. reported a significant difference in MI values between neoplastic tissue and perilesional normal brain tissue (mean values of 25.3% vs. 4.6%), identifying an MI cut-off of 6.8% and demonstrating 88.0% sensitivity, specificity, and accuracy when compared with histopathological evaluation [5]. Similarly, Alexiou et al. (2015) reported significantly lower G0/G1 phase fractions in tumor tissues in patients with GBM and higher S-phase and mitotic fractions in comparison to perilesional tissue, indicating that these parameters may serve as useful markers for intraoperative tumor margin assessment [30]. In another study, Vartholomatos et al. evaluated the utility of the TI and DNA index in assessing glioma margins and reported that both parameters accurately delineated tumor margins [29]. Based on these results, proliferative indices (MI, TI, and cell cycle fractions) provide a reproducible and quantitative approach for assessing tumor margins during surgery and can be used intraoperative for tumor resection.

*Tumor prognosis and recurrence*

IFC has been used to predict prognosis and recurrence risk in patients with intracranial tumors. Vartholomatos et al. reported that GBM patients with G0/G1 < 69% and S-phase > 6% had poorer overall survival [4]. Saito et al. identified an MI cut-off of 26.3% to predict two-year survival with 70% sensitivity and specificity in GBM patients treated with adjuvant radiotherapy and temozolomide. Patients with higher MI values had better prognoses [24].

Oya et al. demonstrated that in schwannoma patients, the PI correlated with MIB-1 and tumor heterogeneity. Tumors with greater heterogeneity, specifically a higher intrameatal PI to cisternal PI ratio (imPI/cPI), were significantly larger and tended to be associated with worse preoperative hearing and a higher risk of recurrence, although the latter was not statistically significant [27]. In meningioma patients, Oya et al. used the same parameter and found that PI correlated with both MIB-1 and the annual growth rate (AGR), indicating that higher proliferative activity predicts faster tumor progression [18].

Suzuki et al. analyzed 102 supratentorial low-grade gliomas and found that diploid tumors were linked to better survival. Aneuploidy was more common in diffuse astrocytomas than in oligodendrogliomas and was associated with progression to GBM [23].

Collectively, these studies demonstrate that proliferative indices and DNA ploidy derived from IFC can serve as surrogate markers for tumor aggressiveness and prognosis.

*Classification of specific tumor subtypes*

IFC has been used both for diagnosing specific tumor types and for differentiating them from others. Alexiou et al. found that large-cell medulloblastomas had lower G0/G1 and higher S-phase and mitotic fractions, correlating with Ki-67. Ependymomas displayed a similar S-phase/Ki-67 correlation and were mostly diploid. Meningiomas, in contrast, showed no significance, with no correlation to Ki-67 or p53; only two were aneuploid. In atypical teratoid/rhabdoid tumors, 58.3% were aneuploid, with no association with Ki-67, p53, or bcl-2 [22].

Vartholomatos et al. reported that primary central nervous system lymphomas (PCNSL) can be identified by the detection of CD markers such as CD45, CD19, or CD20 [28], a finding supported by Alexiou et al., who also identified CD3, CD45, and CD20 expression [32]. Koriyama et al. analyzed 250 glioblastoma and PCNSL cases, noting that PCNSL typically exhibited diploid peaks with low aneuploidy (7.14%) compared to the predominantly aneuploid glioblastomas. Using an S-phase cut-off of 1.42% combined with DNA ploidy, they achieved 89.3% sensitivity, 93.7% specificity, and 93.2% accuracy for differentiating PCNSL from GBM. Although the MI was lower in PCNSL, it lacked diagnostic specificity [24].

Finally, the use of IFC in brain metastatic lesions is limited. Only letters to the editor noted that metastatic brain tumors have a lower G0/G1 fraction and higher S-phase and mitosis fractions than the primary brain tumors [4].

#### 3.4.2. Head and Neck Cancer

Only one prospective study applied IFC in head and neck lesions [6], using cell cycle fractions and the TI. The best cut-off values were G0/G1 < 88% and TI > 10, achieving 97.4% sensitivity and 90% specificity in differentiating neoplastic from non-neoplastic tissue. Moreover, all aneuploid cases were found to be neoplastic [6]. These results demonstrate that even in limited datasets, IFC provides high diagnostic accuracy for intraoperative tumor characterization in head and neck surgery.

#### 3.4.3. Gastrointestinal and Liver Cancer

In this group, three studies explored IFC applications in colorectal, GIST, and liver tumors [10,11,12]. Georvasili et al. (2022) found that TI and cell cycle analysis could accurately differentiate normal from cancerous colorectal tissue and assess rectal cancer margins [10]. A TI cut-off of 10.5% yielded a sensitivity of 82.2% and a specificity of 99.96%, with an overall diagnostic accuracy exceeding 90%. Moreover, they highlighted that the G0/G1 phase fraction varied depending on prior neoadjuvant treatment, suggesting that proliferative activity measured by IFC may reflect treatment response intraoperatively [10].

In liver tumors, IFC was employed in a preliminary study to differentiate primary hepatocellular carcinoma (HCC) from metastatic disease. While HCC tumors typically exhibited diploid status with DNA index = 1 and TI values between 10 and 35%, metastatic lesions showed elevated DNA index more than ≥1.5, indicating aneuploidy [12].

Taniguchi et al. (2021) studied GISTs, using DNA aneuploidy (DA), DNA index (DI), and S-phase fraction (SPF) for calculating the risk of metastasis [11]. They demonstrated that low-risk GISTs were characterized by tumor size ≤ 5 cm, absence of DA, DI < 1.5, and SPF < 2%, whereas intermediate- and high-risk tumors had higher proliferative and aneuploid indices. The overall diagnostic accuracy of IFC in risk stratification ranged from 88.9% to 94.4%.

#### 3.4.4. Breast Cancer and Gynecological Cancer

In breast cancer surgery, IFC was used to assess surgical margins. A prospective study involving 99 patients found that IFC identified 54 positive margins, compared to 14 by standard pathology and 48 by cytology [8]. Using a mitotic index (MI) threshold greater than 5% for diploid tumors, the method achieved 93.3% sensitivity and 92.4% specificity. These tumors also showed decreased G0/G1 fractions and increased S-phase and G2/M activity. Notably, the detection limit for aneuploidy was as low as 0.4–0.8%, highlighting the technique’s high sensitivity [8].

In gynecological cancers, IFC was applied to differentiate normal from malignant tissue. One study analyzing 42 samples reported 100% sensitivity and 90.5% specificity using cut-off values of TI > 6.5% or a G0/G1 fraction below 94.5% [9].

#### 3.4.5. Bladder Cancer

IFC has also been explored in bladder cancer surgery for real-time discrimination between cancer and normal tissue. In a study involving 52 patients, IFC achieved 100% sensitivity and 96.2% specificity using cut-off values of G0/G1 < 93.5% and TI > 6.5%. The diagnostic accuracy was 98.2%. The researchers noted that proliferation analysis and the DNA index of the samples could also assist in evaluating tumor margins [14].

#### 3.4.6. Skin Cancer

In non-melanoma skin cancer, IFC demonstrated similar diagnostic potential comparable to that observed in other types of non-brain tumors. A study of 30 cases assessed tumor margins using cell cycle analysis and the TI. Sensitivity was 95.2% and specificity was 87.1% using cut-off values of TI > 5.75% or a G0/G1 fraction < 94.25%. The overall diagnostic accuracy was 91.1%. Moreover, Markopoulos et al. highlighted that the tumor margins showed higher G0/G1 fractions and lower TI values compared to perilesional tissue [15].

### 3.5. Sensitivity Analysis

A formal sensitivity analysis could not be performed because no quantitative meta-analysis was undertaken. However, the robustness of the synthesized findings was qualitatively assessed by comparing trends across studies with different methodological quality levels and risk-of-bias ratings (as evaluated using the QUADAS-2 tool). Results and conclusions remained consistent when restricting interpretation to studies with low or moderate risk of bias, suggesting that the overall findings are stable and not driven by single studies.

## 4. Discussion

This study is the first systematic review to evaluate the utility, diagnostic performance, and clinical applicability of IFC in tumor surgery. Across 22 included studies encompassing a total of 1511 patients, IFC demonstrated consistent value in real-time intraoperative tumor characterization, margin assessment, and prognostic stratification across a wide spectrum of tumor types—delivering results within just 6 to 10 min.

IFC is a rapid technique that requires only a small tissue sample and is relatively low in cost, as flow cytometers are commonly available in hospitals with cytopathology laboratories. Compared to other real-time intraoperative techniques such as frozen section analysis, intraoperative MRI (iMRI), and fluorescent-guided resection (FGR), IFC offers several practical and logistical advantages. Frozen section analysis, although widely regarded as the gold standard for intraoperative diagnosis, is time-consuming (typically requiring 20 to 62 min for sample processing) and demands the presence of an experienced pathologist [33]. Furthermore, its diagnostic accuracy can be compromised by cautery artifacts, poor tissue quality, and sampling limitations [34]. In contrast, IFC provides objective, quantitative data within minutes, is less affected by tissue handling artifacts, and does not rely on morphological interpretation, making it a more streamlined and reproducible method in real-time surgical settings [2]. Moreover, IFC can analyze both solid and cystic tumor specimens and provides simultaneous information about cell cycle distribution, DNA ploidy, and proliferative activity—parameters that cannot be assessed by frozen section biopsy. FGR using 5-aminolevulinic acid (5-ALA) is currently the only FDA-approved method for GBM surgery [35]. However, its use is limited to specific tumor types, and its effectiveness in recurrent tumors or non-GBM lesions remains restricted. In addition, the high cost of 5-ALA, limited tissue penetration of the fluorescent signal, and variability in intraoperative visualization present significant challenges [36]. In contrast, IFC is applicable to a broader range of tumors and does not rely on specific metabolic or optical properties of the tissue [2]. iMRI enables real-time detection of residual tumors and has been associated with higher rates of gross total resection and improved outcomes in certain tumor types [37]. Nevertheless, its use is associated with extremely high costs, the requirement for specialized MRI-compatible operating rooms and equipment, prolonged operative time, and limited availability in high-resource centers only [37,38]. By comparison, IFC can be seamlessly integrated into existing surgical workflows with minimal setup, and results are typically available within 5 to 10 min, making it a highly accessible and practical alternative (Table 6) [2]. However, it lacks information on tissue morphology, requires fresh samples and trained personnel, and currently lacks standardized protocols for solid tumors. Despite these limitations, its rapid learning curve, the fact that it only requires a flow cytometer which is available in most hospital laboratories, and the low cost of reagents and antibodies make it a practical, accessible, and cost-effective tool for real-time tumor characterization during surgery [17].

Based on the results of this systematic review, IFC has been most widely applied in intracranial tumors, particularly gliomas and meningiomas, where it has proven effective in grading tumors, delineating surgical margins, and predicting recurrence risk [4,5,18,25,26,29,30,31]. Across multiple studies, cell cycle fractions (G0/G1, S-phase, G2/M, MI, PI, TI, and DNA ploidy status) have been used as key IFC parameters for real-time tumor characterization. Sensitivity and specificity for tumor grading and margin identification varied but were generally high, ranging from 80% to 93.3% and 88% to 100%, respectively [5,8,10,30,31]. In glioma surgery, G0/G1 and S-phase cut-off values enabled discrimination between low-grade and high-grade gliomas with sensitivities up to 94.1% and specificities up to 100% [4,30,32]. Notably, some studies reported the prognostic utility of IFC-derived indices, correlating proliferative activity and ploidy status with survival outcomes [4,18,23,26,27].

Furthermore, the application of IFC in immunophenotyping using CD markers introduces new opportunities for immediate intraoperative diagnosis of lymphoproliferative disorders such as primary central nervous system lymphoma (PCNSL) [28,32]. The identification of markers like CD20 has direct therapeutic implications, including the potential initiation of targeted therapies such as rituximab [39].

Beyond the central nervous system, IFC has also shown promising results in various extracranial malignancies. In head and neck cancers, IFC distinguished neoplastic from benign tissue with 97.4% sensitivity and 90% specificity using G0/G1 and TI values [6]. In gastrointestinal tumors and especially colorectal cancer, IFC was able to identify cancerous tissue and assess margins with over 90% diagnostic accuracy [11]. Applications in breast, gynecological, bladder, liver, and skin cancers further support the cross-specialty relevance of IFC, with high sensitivity, specificity, and real-time interpretability enhancing its intraoperative utility. In breast cancer specifically, the rapid evaluation of margins with IFC holds the potential to improve surgical outcomes and patient satisfaction while reducing the burden of repeat surgeries [8]. In liver cancer, its use remains preliminary, and further studies are needed to evaluate its effectiveness [12].

### 4.1. Risk of Bias and Certainty of Evidence

The risk of bias due to missing results or selective reporting was evaluated qualitatively. Because most included studies were small, single-center reports, publication bias cannot be excluded. However, all retrieved studies that met the inclusion criteria were incorporated, and no evidence of selective outcome reporting within individual studies was identified. The absence of unpublished or non-English studies, as well as the exclusion of gray literature, may have introduced minor reporting bias, which is acknowledged as a limitation of this review.

The overall certainty of the evidence supporting intraoperative flow cytometry (IFC) as a diagnostic and prognostic tool in surgical oncology was rated as *moderate to low*. Confidence was downgraded due to (i) the predominance of small observational studies, (ii) heterogeneity in IFC methodology and reporting, and (iii) limited external validation across tumor types. Nevertheless, consistent diagnostic accuracy ranges and reproducible cut-off trends across independent studies, particularly in brain tumors, support moderate confidence in the direction and magnitude of the observed effects.

### 4.2. Limitations

Despite the comprehensive nature of this review, several limitations should be acknowledged. First, there is substantial heterogeneity in study designs, protocols, and outcome measures, which limit the generalizability of findings. Cut-off values for cell cycle fractions and indices varied across studies, likely due to differences in sample processing methods, tumor biology, and staining protocols.

Regarding the clinical application of IFC, the existing literature is limited for certain tumor types and clinical scenarios. IFC appears to be most reliably used for distinguishing low- from high-grade gliomas or meningiomas and for identifying glioma margins—areas where most of the data are concentrated. However, in specific cancer types such as pediatric brain tumors, brain metastases, primary central nervous system lymphomas (PCNSL), and liver tumors, the findings are limited or preliminary and require further validation in larger, prospective cohorts.

Moreover, our search was restricted to three databases. Although the search strategy was designed to capture all relevant keywords related to IFC in tumor surgery, it is possible that studies published in other databases or in languages other than English were missed. While we included some letters to the editor that presented original data, we excluded others without original findings, and we did not include studies from gray literature, which may have further limited the scope of our findings.

Another limitation involves the design and quality of the studies included. While many were prospective, several were retrospective or lacked detailed methodological reporting, introducing a risk of bias. According to QUADAS-2 assessments, several studies showed high or unclear risk of bias, particularly in patient selection and interpretation of the index test, which could affect the reported diagnostic performance.

An additional limitation of this review is the inclusion of all tumor categories, despite the limited number of studies available for certain cancer types. This decision was intentional, aiming to provide a comprehensive overview of IFC applications across surgical oncology. However, the small number of studies within specific tumor subgroups restricts the ability to perform statistical pooling or subgroup meta-analyses. Consequently, the strength of conclusions for these tumor types is limited. Nonetheless, presenting the entire current landscape of IFC research offers valuable insights and serves as a foundation for future multicenter investigations and standardized clinical protocols. Finally, many of the included studies enrolled a relatively small number of patients, often fewer than 20 participants, which reduces statistical power and limits the robustness and generalizability of their conclusions. Moreover, most studies originate from the same research groups, which may limit methodological diversity and hinder the establishment of standardized protocols for intraoperative flow cytometry.

### 4.3. Future Directions

IFC is a rapid, real-time intraoperative diagnostic tool for guiding tumor resection; however, its widespread clinical application has yet to be realized [2]. As highlighted in this systematic review, the current data are limited and heterogeneous, both in methodology and in cut-off values, particularly in non-brain tumors.

For broader clinical adoption, it is necessary to establish standardized protocols, conduct validation studies across various tumor types, and integrate IFC into routine surgical workflows. Researchers should prioritize exploring the use of IFC in tumor types where data are currently lacking, such as pancreatic, lung, liver, and kidney cancers. Additionally, there is a need to define consistent cut-off values for tumor index (TI), mitotic or malignancy index (MI), and cell cycle fractions in tumor types where IFC has already been applied.

Furthermore, collaborative, multi-institutional studies with larger sample sizes are essential to confirm the diagnostic and prognostic utility of IFC across diverse malignancies. Importantly, integrating IFC with other intraoperative tool could provide synergistic benefits and further enhance surgical precision.

Finally, the use of artificial intelligence (AI) and machine learning algorithms may further expand the role of IFC in real-time oncologic surgery [40]. By leveraging large datasets generated from flow cytometric parameters such as cell cycle fractions, DNA ploidy, TI, and MI. This integration could reduce diagnostic subjectivity, optimize intraoperative decision-making, and support the development of personalized surgical strategies. In the future, AI-enhanced IFC systems may become key components of intelligent surgical platforms, driving improvements in accuracy, efficiency, and outcomes in oncologic care.

In terms of practical implementation, the cost of IFC is relatively low, and the necessary equipment is already available in most laboratories. Training requirements are minimal, with proficiency achievable through short, blended programs combining theoretical instruction and hands-on practice. These factors, together with standardization and technological integration, suggest that widespread adoption of IFC in routine oncology practice is both feasible and cost-effective once standardized protocols are established.

## 5. Conclusions

Intraoperative flow cytometry represents a valuable, rapid, and real-time tool for characterizing tumor grade, margins, and subtype across a range of tumor types, particularly in the neurosurgical setting. This method shows great promise in supporting intraoperative decision-making and informing patient prognosis in surgical oncology.

However, to establish its use in everyday clinical practice and potentially as an alternative to frozen section biopsies, further research is required. Specifically, large-scale validation studies and the development of standardized protocols are essential to ensure that IFC can be effectively and reliably integrated into routine surgical workflows.

## Figures and Tables

**Table 1:** Characteristics of the included studies.

Ref.	Study	Year	Type of Study	Object	No of Patients	IFC Parameter
[5]	Shioyama et al.	2013	Prospective	Brain Tumors	81	Malignant Index (MI)
[4]	Vartholomatos et al.	2013	n/a	Brain Tumors	63	Cell Cycle Fractions
[22]	Alexiou et al.	2015	Retrospective	Pediatric Brain Tumors	68	Cell Cycle fractions
[30]	Alexiou et al.	2015	Prospective	Brain Tumors	31	Cell Cycle Fractions
[23]	Suzuki et al.	2018	Retrospective	Brain Tumors	102	Aneuploidy
[24]	Koriyama et al.	2018	Retrospective	Brain Tumors	250	MI
[25]	Matsuoka et al.	2018	Prospective	Brain Tumors	117	MI
[28]	Vartholomatos et al.	2018	n/a	Brain Tumors	5	CD Markers
[18]	Oya et al.	2019	Prospective	Brain Tumors	50	Proliferation Index (PI)
[26]	Saito et al.	2019	Retrospective	Brain Tumors	102	MI
[27]	Oya et al.	2022	Prospective	Brain Tumors	34	PI
[31]	Vartholomatos et al.	2023	Retrospective	Brain Tumors	81	Tumor Index (ΤΙ)
[32]	Alexiou et al.	2023	Retrospective	Brain Tumors	59	ΤΙ
[29]	Alexiou, Markopoulos et al.	2023	n/a	Brain Tumors	n/a	CD Markers
[6]	Vartholomatos et al.	2019	Prospective	Head and Neck Cancer	70	Cell Cycle Fractions TI
[10]	Georvasili et al.	2022	Prospective	Colorectal Cancer	106	ΤΙ Cell Cycle Fraction
[12]	Markopoulos et al.	2021	n/a	Primary and Metastatic Liver Neoplasms	9	TI DNA Index (DI)
[11]	Taniguchi et al.	2021	Retrospective	Gastrointestinal Stromal Tumors (GIST)	18	DNA Aneuploidy (DA) DI S-phase fraction (SPF)
[8]	Vartholomatos et al.	2021	Prospective	Breast Cancer	99	MI Cell Cycle Fractions
[9]	Anastasiadi et al.	2022	Retrospective	Gynecological Cancer	42 (50 but 8 samples have been excluded)	Cell Cycle Fractions TI
[14]	Paliouras et al.	2022	Prospective	Bladder Cancer Normal	52	Cell Cycle Fractions TI
[15]	Markopoulos et al.	2024	Prospective	Non-Melanoma Skin Cancer	30	Cell Cycle Fractions TI

**Table 2:** Intraoperative flow cytometry for tumor grading and differential diagnosis among various tumor types.

Study	Year	Object	No of Patients	IFC Parameter	Sensitivity	Specificity	Cut-Off	Outcome	Comments
Shioyama et al. [5]	2013	Glioma grade	81	Malignant Index (MI)	n/a	n/a	MI: 6.9%	Lower MI in LGG	-
Vartholomatos et al. [4]	2013	LGG vs. HGG	63	Cell Cycle Fractions	82.2% 94.1%	100% 100%	G0/G1: 78% S phase: 4.3%	High accuracy	Letter to Editor
		Meningiomas			n/a	n/a	n/a	Grade indication based on cell cycle	
		Brain metastasis			n/a	n/a	Low G0/G1, High S phase and high G2/M	Primary vs. metastasis	
Alexiou et al. [22]	2015	Grade I/II vs. III/IV	68	Cell Cycle Fractions	80%	88.9%	G0/G1: 81%	Grading	Pediatric population
Alexiou et al. [30]	2015	LGG vs. HGG	31	Cell Cycle Fractions	92.3% 84.6% 92.3%	91.7% 91.7% 91.7%	G0/G1: 75% S: 6% Mitosis: 9.7%	Grading	Optimal cut-off G0/G1 75%
Koriyama et al. [24]	2018	GBM vs. PCNSL	250	MI	89.3%/	93.7%	S1: 6.24% and S2: 1.42%	Strong separation of PCNSL vs. GBM	
Matsuoka et al. [25]	2018	Meningiomas Grade	117	MI	64.7%	85%	MI: 8%	Grading	Asian patients
Vartholomatos et al. [28]	2018	PCNSL vs. GBM	5	CD	n/a	n/a	CD45/CD29/CD 20 in PCNSL	Immunotyping	Letter to Editor
Vartholomatos et al. [31]	2023	LGG vs. HGG Glioma Margins	81	Tumor Index	61.4%	100%	17%	All aneuploid tumors = HGG	Useful for assessing tumor grade and extent of infiltration
Alexiou et al. [32]	2023	Meningiomas Low vs. High Grade	59	Tumor Index	90.2%	72.2%	TI: 11.4%	High-grade → higher S phase and Mitosis and lower G0/G1 phase fraction	Good correlation
Alexiou, Markopoulos et al. [29]	2023	PCNSL	n/a	CD markers	n/a	n/a	CD 3/CD 45/CD 20 detection	PCNSL diagnosis	Letter to Editor
Vartholomatos et al. [6]	2019	Head and Neck Cancer Benign vs. neoplastic	70	Cell cycle fractions	97.4% 97.4% 80% 97.4%	90% 73.3% 86.7% 90%	G0/G1: 88% S phase: 6% G2/M: 5% TI > 10%	Characterization of head and neck lesions	All aneuploid lesions were neoplastic
Markopoulos et al. [12]	2021	Primary and Metastatic Liver Neoplasms	9	Tumor Index (TI) DNA Index	n/a	n/a	HCC → DNA index of 1 and tumor index of 10–35% In metastatic disease → DNA index ≥1.5, indicative aneuploidy	Cell characterization and margin detection during the excision Primary vs. Metastatic lesion	Study protocol—preliminary study

**Table 3:** Intraoperative flow cytometry for assessing tumor margins and differentiating normal from cancerous tissue.

Study	Year	Object	No of Patients	IFC Parameter	Sensitivity	Specificity	Cut-Off	Outcome	Comments
Shioyama et al. [5]	2013	Gliomas margins	81	Malignant Index (MI)	88%	88%	MI: 6.9%	Good discrimination between tumor and normal tissue	MI higher in neoplastic tissue
Alexiou et al. [22]	2015	Neoplastic vs. normal	68	Cell Cycle fractions	100% 100% 98.4%	100% 100% 100%	G0/G1: 98% Mitosis ≤ 2% S phase: 2.2%	Excellent accuracy	Pediatric population
Alexiou et al. [30]	2015	GBM margins	31	Cell Cycle Fractions	n/a	n/a	Lower G0/G1 and higher S phase fractions	Margins	-
Georvasili et al. [10]	2022	Colorectal cancer Normal vs. cancer tissue	106	Tumor Index (TI)	82.2%	99.96%	TI cut-off 10.5%	Accuracy of TI > 90%	TI is linked with the tumor stage (lower in advanced stages)
		Rectal cancer margins		Cell Cycle Fraction	n/a	n/a	G0/G1 for normal, cancer without neoadjuvant therapy and cancer samples with neoadjuvant therapy were 93.51%, 73.75%, and 87.08%	Accuracy of IFC was 79%, 88% and 85%, respectively; G0/G1 showed significant difference between normal and cancer samples	Neoadjuvant therapy reduced cancer cell replication
Vartholomatos et al. [8]	2021	Breast cancer margins	99	Malignancy Index (MI) Cell Cycle Fractions	93.3%	92.4	In diploid MI cutoff >5% Mean tumor fractions: G0/G1 77.3%, S 7.3%, G2/M 15.3%, MI 22.6%. Diploid tumors: ↓ G0/G1, ↑ G2/M, ↑ MI	Accuracy of IFC: 92.5% IFC identified 54/506 positive margins vs. 14 by pathology and 48 cytology Aneuploid detection limit: 0.4–0.8%	IFC offer a rapid evaluation of the margins in breast cancer surgery
Anastasiadi et al. [9]	2022	Gynecological cancer Normal vs. cancer tissue	42 (50 but 8 samples have been excluded)	Cell Cycle Fractions TI	100%	90.5%	G0/G1 < 94.5% or TI > 6.5%	100% accuracy	-
Paliouras et al. [14]	2022	Bladder cancer Normal vs. cancer tissue	52	Cell Cycle Fractions TI	100%	96.2%	G0/G1 < 93.5% or TI > 6.5%	98.2% accuracy PPV 96.2% NPV 100%	DNA index and proliferation analysis support tumor margin assessment
Markopoulos et al. [15]	2024	Non-Melanoma Skin Cancer	30	Cell Cycle Fractions TI	95.2%	87.1%	G0/G1 < 94.25% or TI > 5.75%	91.1% accuracy	Higher G0/G1 fraction and lower TI in tumor margins

↓: Decreased number of cells/low number of cells; ↑: Increased number of cells/high number of cells.

**Table 4:** Intraoperative flow cytometry in the evaluation of tumor prognosis, risk of recurrence, and metastatic potential.

Study	Year	Object	No of Patients	IFC Parameter	Sensitivity	Specificity	Cut-Off	Outcome	Comments
Vartholomatos et al. [4]	2013	GBM prognosis	n/a (number of GBM patients not specified)	Cell Cycle Fractions	n/a	n/a	Lower survival with G0/G1 ≤ 69% S phase > 6%	Predicts poor survival	Letter to Editor
Suzuki et al. [23]	2018	LGG	102	Aneuploidy	n/a	n/a	Aneuploidy tumors have more aggressive behavior	Aneuploidy = poor prognosis	DNA ploidy as prognostic factor in LGG and especially in DA
Oya et al. [18]	2019	Meningiomas	50	PI	n/a	n/a	PI correlated with MIB-1. Higher PI → higher AGR and more pial feeders	Proliferation Heterogeneity	PI location varied with AGR. High ACR → peaked in center or periphery. Low ACR → PI peaked in dural attachment
Saito et al. [19]	2019	GBM prognosis	102	MI	70%	70%	2 years survival cut-off vs. no 2 years survival MI 26.3%	Better survival with MI ≥ 26.3%	Linked to IDH1 status. MI greater impact on patient with IDH1 wild type
Oya et al. [27]	2022	Vestibular Schwannoma	34	Proliferation Index (PI)	n/a	n/a	imPI/cPI ratio	Heterogeneity Analysis High imPI/cPI linked to larger size, recurrence	Procedure ~10 min, MIB-1 correlated (R = 0.57) Not significant
Taniguchi [11]	2021	GIST metastatic risk	18	DNA aneuploidy (DA) DNA index (DI) S-phase fraction (SPF)	Low risk → 100% Intermediate risk GIST→ 71.4%, High-risk GIST → 100%	Low risk → 87.5% Intermediate risk GIST→ 100%, High-risk GIST → 94.1%	Calculate the metastatic risk of GIST with DA, DI SPF	Low risk size ≤ 5 cm, absence of DA and DI < 1.5 and SPF < 2 Intermediate risk size ≤ 5 cm, presence of DA or DI ≥ 1.5 or SPF ≥ 2 or size between 5.1 and 10 cm, absence of DA and DI < 1.5 and SPF < 2	Accuracy from 88.9 to 94.4% IFC and Tumor Size → Correlation with the modified Fletcher risk classification.

**Table 5:** Cut-off values of intraoperative flow cytometry (IFC) parameters for each tumor type.

Tumor Type	Study/Year	IFC Parameter(s)	Cut-Off/Threshold Value(s)	Interpretation/Application
**Low- vs. High-Grade Gliomas (LGG/HGG)**	Shioyama 2013 [5]	Malignancy Index (MI)	MI 6.9%	Higher MI distinguishes tumor from normal tissue
	Vartholomatos 2013 [4]	Cell cycle fractions	G0/G1 78% S phase 4.3%	Distinguishes LGG vs. HGG
	Alexiou 2015 [30]	Cell cycle fractions	G0/G1 75% S phase 6% MITOSIS 9.7%	Grading accuracy > 90%
	Vartholomatos 2023 [31]	Tumor Index (TI)	TI 17%	All aneuploid tumors = HGG; useful for grade and infiltration
**Glioblastoma (GBM)**	Saito 2019 [26]	MI	26.3%	MI ≥ 26.3% associated with better 2-year survival (IDH1-dependent)
	Koriyama 2018 [24]	S1, S2 cell cycle fractions	S1 6.24% S2 1.42%	Distinguishes GBM vs. PCNSL
	Vartholomatos 2013 [4]	Cell cycle fractions Lower survival	G0/G1 ≤ 69% S phase > 6%	Predicts poor survival lower survival
**Meningiomas**	Matsuoka 2018 [25]	MI	MI 8%	Differentiates low- vs. high-grade tumors
	Alexiou 2023 [32]	TI	TI 11.4%	High-grade → higher S and M phases, lower G0/G1
**Primary CNS Lymphoma (PCNSL)**	Vartholomatos 2018 [28] Alexiou 2023 [32]	CD markers	—	Diagnostic immunophenotyping (CD45, CD20, CD29)
**Vestibular Schwannoma**	Oya 2022 [27]	imPI/cPI ratio	—	High ratio → larger size, recurrence risk
**Head and Neck Tumors**	Vartholomatos 2019 [6]	Cell cycle fractions TI	G0/G1 88% S phase 6% G2/M 5% TI > 10%	Distinguishes benign vs. malignant lesions
**Colorectal Cancer**	Georvasili 2022 [10]	TI Cell cycle fractions	TI 10.5%; G0/G1 ≈ 74–93%	Differentiates normal, cancer, and neoadjuvant-treated tissue
**Liver Tumors (HCC/Metastases)**	Markopoulos 2021 [12]	TI DNA Index (DI)	HCC TI 10–35%; DI = 1 (normal)/≥ 1.5 (metastatic)	Differentiates primary vs. metastatic lesions
**Gastrointestinal Stromal Tumor (GIST)**	Taniguchi 2021 [11]	DA, DI, SPF	DI < 1.5 and SPF < 2 → low risk DI ≥ 1.5 or SPF ≥ 2 → higher risk	Correlates with modified Fletcher risk classification
**Breast Cancer**	Vartholomatos 2021 [8]	MI Cell cycle fractions	MI > 5% G0/G1 77% S phase 7% G2/M 15%	Rapid margin assessment; detects aneuploidy ≥ 0.4%
**Gynecologic Tumors**	Anastasiadi 2022 [9]	G0/G1 TI	G0/G1 < 94.5% or TI > 6.5%	Differentiates normal vs. malignant tissue
**Bladder Cancer**	Paliouras 2022 [14]	G0/G1 TI	G0/G1 < 93.5% or TI > 6.5%	98% accuracy for tumor margin assessment
**Non-Melanoma Skin Cancer**	Markopoulos 2024 [15]	G0/G1 TI	G0/G1 < 94.25% or TI > 5.75%	91% accuracy higher G0/G1 fraction in tumor margins

**Table 6:** Intraoperative flow cytometry and other intraoperative techniques.

Technique	Diagnostic Principle	Average Time to Result	Main Advantages	Limitations	Approximate Cost/Accessibility	Applicable Tumor Types
Intraoperative Flow Cytometry (IFC)	Quantitative cell analysis using DNA content, ploidy, and cell cycle distribution	5–10 min	Rapid analysis Quantitative results Small sample required Detects ploidy and proliferative Indices Reproducible and easily integrated into workflow	Requires flow cytometer and trained operator Limited reference standards for non-brain tumors No available protocols and standards cut-off values	Low (uses existing cytometry equipment in most centers)	Brain, head and neck, colorectal, breast, liver, gynecologic, bladder, skin, pancreatic
Frozen Section Analysis	Histological evaluation of cryosectioned tissue under light microscopy	20–60 min	Established “gold standard” for intraoperative diagnosis Provides morphological detail	Time-consuming Sampling errors Requires experienced pathologist Artifacts from cautery or freezing	Moderate (requires pathology lab and expertise)	Most tumor types
Fluorescence-Guided Resection (FGR, 5-ALA)	Tumor visualization via selective accumulation of 5-ALA metabolite (PpIX) under blue light	Require administration 3 h before the surgery	Real-time visualization of tumor margins Improve resection in GBM and HGG surgery	Limited to specific CNS tumor types Costly (EUR 1000 every dose in Europe) Variable fluorescence intensity	High (cost of 5-ALA and optical systems)	High-grade gliomas Other types of CNS tumors without FDA approval and mixed results
Intraoperative MRI (iMRI)	Real-time MRI imaging of residual tumor during surgery	>30–90 min	Real-time volumetric assessment; improves gross total resection	Very high cost Requires specialized equipment Prolongs surgery time Limited availability	Very High (infrastructure-intensive)	Brain, spine Can be used in all types of tumors

## Data Availability

Data is provided within the manuscript or in the Supplementary Material.

## Supplementary Materials

The following supporting information can be downloaded at: https://www.mdpi.com/article/10.3390/cancers17243898/s1, Table S1: PRISMA 2020 checklist.

## Abbreviations

5-ALA	5-Aminolevulinic acid
AGR	Annual growth rate
AUC	Area under the ROC curve
CD	Cluster of differentiation
CD3-FITC	CD3 antigen labeled with fluorescein isothiocyanate
CNS	Central nervous system
DA	DNA aneuploidy
DI	DNA index
DNA	Deoxyribonucleic acid
FC	Flow cytometry
FDA	Food and Drug Administration
FGR	Fluorescence-guided resection
G0/G1	Gap 0/Gap 1 phases of the cell cycle
G2/M	Gap 2/Mitosis phases of the cell cycle
GBM	Glioblastoma
GFP	Green fluorescent protein
GIST	Gastrointestinal stromal tumor
HCC	Hepatocellular carcinoma
HGG	High-grade glioma
IDH1	Isocitrate dehydrogenase 1
IFC	Intraoperative flow cytometry *(preferred form used throughout)*
iMRI	Intraoperative magnetic resonance imaging
Ki-67/MIB-1	Proliferation marker (MIB-1 antibody against Ki-67)
LGG	Low-grade glioma
MI	Malignancy or mitotic index
n/a	not available/applicable
NPV	Negative predictive value
PBMC	Peripheral blood mononuclear cells
PCNSL	Primary central nervous system lymphoma
PI	Proliferation index
PPV	Positive predictive value
PRISMA	Preferred Reporting Items for Systematic Reviews and Meta-Analyses
QUADAS-2	Quality Assessment of Diagnostic Accuracy Studies 2
S-phase	DNA synthesis phase of the cell cycle
SPF	S-phase fraction
TI	Tumor index *(combined S-phase + G2/M fractions)*
imPI/cPI	Intrameatal PI to cisternal PI ratio

## References

[1] McKinnon K.M. (2018). Flow Cytometry: An Overview. Curr. Protoc. Immunol..

[2] Liaropoulos I., Liaropoulos A., Liaropoulos K. (2023). Critical Assessment of Cancer Characterization and Margin Evaluation Techniques in Brain Malignancies: From Fast Biopsy to Intraoperative Flow Cytometry. Cancers.

[3] Kocarnik J.M., Compton K., Dean F.E., Fu W., Gaw B.L., Harvey J.D., Henrikson H.J., Lu D., Pennini A., Global Burden of Disease 2019 Cancer Collaboration (2022). Cancer Incidence, Mortality, Years of Life Lost, Years Lived with Disability, and Disability-Adjusted Life Years for 29 Cancer Groups From 2010 to 2019: A Systematic Analysis for the Global Burden of Disease Study 2019. JAMA Oncol..

[4] Vartholomatos G., Alexiou G.A., Batistatou A., Kyritsis A.P., Voulgaris S. (2013). Intraoperative diagnosis. J. Neurosurg..

[5] Shioyama T., Muragaki Y., Maruyama T., Komori T., Iseki H. (2013). Intraoperative flow cytometry analysis of glioma tissue for rapid determination of tumor presence and its histopathological grade: Clinical article. J. Neurosurg..

[6] Vartholomatos G., Basiari L., Exarchakos G., Kastanioudakis I., Komnos I., Michali M., Markopoulos G.S., Batistatou A., Papoudou-Bai A., Alexiou G.A. (2019). Intraoperative flow cytometry for head and neck lesions. Assessment of malignancy and tumour-free resection margins. Oral Oncol..

[7] Kastanioudakis I., Basiari L. (2023). Intraoperative Flow Cytometry in Head and Neck Malignancies.

[8] Vartholomatos G., Harissis H., Andreou M., Tatsi V., Pappa L., Kamina S., Batistatou A., Markopoulos G.S., Alexiou G.A. (2021). Rapid Assessment of Resection Margins During Breast Conserving Surgery Using Intraoperative Flow Cytometry. Clin. Breast Cancer.

[9] Anastasiadi Z., Mantziou S., Akrivis C., Paschopoulos M., Balasi E., Lianos G.D., Alexiou G.A., Mitsis M., Vartholomatos G., Markopoulos G.S. (2022). Intraoperative Flow Cytometry for the Characterization of Gynecological Malignancies. Biology.

[10] Georvasili V.K., Markopoulos G.S., Batistatou A., Mitsis M., Messinis T., Lianos G.D., Alexiou G., Vartholomatos G., Bali C.D. (2022). Detection of cancer cells and tumor margins during colorectal cancer surgery by intraoperative flow cytometry. Int. J. Surg..

[11] Taniguchi K., Suzuki A., Serizawa A., Kotake S., Ito S., Suzuki K., Yamada T., Noguchi T., Amano K., Ota M. (2021). Rapid Flow Cytometry of Gastrointestinal Stromal Tumours Closely Matches the Modified Fletcher Classification. Anticancer Res..

[12] Markopoulos G.S., Glantzounis G.K., Goussia A.C., Lianos G.D., Karampa A., Alexiou G.A., Vartholomatos G. (2021). Touch Imprint Intraoperative Flow Cytometry as a Complementary Tool for Detailed Assessment of Resection Margins and Tumor Biology in Liver Surgery for Primary and Metastatic Liver Neoplasms. Methods Protoc..

[13] Lehman E. (2022). Utilizing Intraoperative Flow Cytometry to Accurately Characterize Bladder Cancer Cells. Surg. Curr. Res..

[14] Paliouras A., Markopoulos G.S., Tsampalas S., Mantziou S., Giannakis I., Baltogiannis D., Glantzounis G.K., Alexiou G.A., Lampri E., Sofikitis N. (2022). Accurate Characterization of Bladder Cancer Cells with Intraoperative Flow Cytometry. Cancers.

[15] Markopoulos G., Lampri E., Tragani I., Kourkoumelis N., Vartholomatos G., Seretis K. (2024). Intraoperative Flow Cytometry for the Rapid Diagnosis and Validation of Surgical Clearance of Non-Melanoma Skin Cancer: A Prospective Clinical Feasibility Study. Cancers.

[16] Alexiou G.A., Vartholomatos G., Kobayashi T., Voulgaris S., Kyritsis A.P. (2020). The emerging role of intraoperative flow cytometry in intracranial tumor surgery. Clin. Neurol. Neurosurg..

[17] Quirós-Caso C., Arias Fernández T., Fonseca-Mourelle A., Torres H., Fernández L., Moreno-Rodríguez M., Ariza-Prota M.Á., López-González F.J., Carvajal-Álvarez M., Alonso-Álvarez S. (2022). Routine flow cytometry approach for the evaluation of solid tumor neoplasms and immune cells in minimally invasive samples. Cytometry B Clin. Cytom..

[18] Oya S., Yoshida S., Tsuchiya T., Fujisawa N., Mukasa A., Nakatomi H., Saito N., Matsui T. (2019). Intraoperative quantification of meningioma cell proliferation potential using rapid flow cytometry reveals intratumoral heterogeneity. Cancer Med..

[19] Page M.J., McKenzie J.E., Bossuyt P.M., Boutron I., Hoffmann T.C., Mulrow C.D., Shamseer L., Tetzlaff J.M., Akl E.A., Brennan S.E. (2021). The PRISMA 2020 statement: An updated guideline for reporting systematic reviews. BMJ.

[20] Reitsma J.B., Rutjes A.W., Whiting P., Yang B., Leeflang M.M., Bossuyt P.M., Deeks J.J., Deeks J.J., Bossuyt P.M., Leeflang M.M., Takwoingi Y. (2023). Assessing Risk of Bias and Applicability. Cochrane Handbook for Systematic Reviews of Diagnostic Test Accuracy.

[21] Whiting P.F., Rutjes A.W.S., Westwood M.E., Mallett S., Deeks J.J., Reitsma J.B., QUADAS-2 Group (2011). QUADAS-2: A revised tool for the quality assessment of diagnostic accuracy studies. Ann. Intern. Med..

[22] Alexiou G.A., Vartholomatos G., Stefanaki K., Lykoudis E.G., Patereli A., Tseka G., Tzoufi M., Sfakianos G., Prodromou N. (2015). The Role of Fast Cell Cycle Analysis in Pediatric Brain Tumors. Pediatr. Neurosurg..

[23] Suzuki A., Maruyama T., Nitta M., Komori T., Ikuta S., Chernov M., Tamura M., Kawamata T., Muragaki Y. (2018). Evaluation of DNA ploidy with intraoperative flow cytometry may predict long-term survival of patients with supratentorial low-grade gliomas: Analysis of 102 cases. Clin. Neurol. Neurosurg..

[24] Koriyama S., Nitta M., Shioyama T., Komori T., Maruyama T., Kawamata T., Muragaki Y. (2018). Intraoperative Flow Cytometry Enables the Differentiation of Primary Central Nervous System Lymphoma from Glioblastoma. World Neurosurg..

[25] Matsuoka G., Eguchi S., Anami H., Ishikawa T., Yamaguchi K., Nitta M., Muragaki Y., Kawamata T. (2018). Ultrarapid Evaluation of Meningioma Malignancy by Intraoperative Flow Cytometry. World Neurosurg..

[26] Saito T., Muragaki Y., Shioyama T., Komori T., Maruyama T., Nitta M., Yasuda T., Hosono J., Okamoto S., Kawamata T. (2019). Malignancy Index Using Intraoperative Flow Cytometry is a Valuable Prognostic Factor for Glioblastoma Treated With Radiotherapy and Concomitant Temozolomide. Neurosurgery.

[27] Oya S., Yoshida S., Hanakita S., Inoue M. (2022). Quantitative Evaluation of Proliferative Potential Using Flow Cytometry Reveals Intratumoral Heterogeneity and Its Relevance to Tumor Characteristics in Vestibular Schwannomas. Curr. Oncol..

[28] Vartholomatos G., Alexiou G.A., Voulgaris S., Kyritsis A.P. (2018). Intraoperative Immunophenotypic Analysis for Diagnosis and Classification of Primary Central Nervous System Lymphomas. World Neurosurg..

[29] Alexiou G.A., Markopoulos G., Voulgaris S., Vartholomatos G. (2023). Usefulness of intraoperative rapid flow cytometry in the surgical treatment of brain tumors. Neuropathology.

[30] Alexiou G.A., Vartholomatos G., Goussia A., Batistatou A., Tsamis K., Voulgaris S., Kyritsis A.P. (2015). Fast cell cycle analysis for intraoperative characterization of brain tumor margins and malignancy. J. Clin. Neurosci..

[31] Vartholomatos G., Markopoulos G.S., Vartholomatos E., Goussia A.C., Dova L., Dimitriadis S., Mantziou S., Zoi V., Nasios A., Sioka C. (2023). Assessment of Gliomas’ Grade of Malignancy and Extent of Resection Using Intraoperative Flow Cytometry. Cancers.

[32] Alexiou G.A., Markopoulos G.S., Vartholomatos E., Goussia A.C., Dova L., Dimitriadis S., Mantziou S., Zoi V., Nasios A., Sioka C. (2023). Intraoperative Flow Cytometry for the Evaluation of Meningioma Grade. Curr. Oncol..

[33] Jaafar H. (2006). Intra-operative frozen section consultation: Concepts, applications and limitations. Malays. J. Med. Sci..

[34] Amit S., Khan L., Chakraborty S., Pai M.R., Naik R. (2024). Diagnostic Utility, Errors and Limitations of Frozen Section in Surgical Pathology. Int. J. Acad. Med. Pharm..

[35] Hadjipanayis C.G., Stummer W. (2019). 5-ALA and FDA approval for glioma surgery. J. Neurooncol..

[36] Rybaczek M., Chaurasia B. (2024). Advantage and challenges in the use of 5-Aminolevulinic acid (5-ALA) in neurosurgery. Neurosurg. Rev..

[37] Madani D., Fonseka R.D., Kim S.J., Tang P., Muralidharan K., Chang N., Wong J. (2025). Comparing the Rates of Further Resection After Intraoperative MRI Visualisation of Residual Tumour Between Brain Tumour Subtypes: A 17-Year Single-Centre Experience. Brain Sci..

[38] Roder C., Haas P., Tatagiba M., Ernemann U., Bender B. (2021). Technical limitations and pitfalls of diffusion-weighted imaging in intraoperative high-field MRI. Neurosurg. Rev..

[39] Smith M.R. (2003). Rituximab (monoclonal anti-CD20 antibody): Mechanisms of action and resistance. Oncogene.

[40] Koriyama S., Matsui Y., Shioyama T., Onodera M., Tamura M., Kobayashi T., Ro B., Masui K., Komori T., Muragaki Y. (2025). High-precision intraoperative diagnosis of gliomas: Integrating imaging and intraoperative flow cytometry with machine learning. Front. Neurol..

